# Automation and Optimization of Rat Heart Decellularization Using a Vibrating Fluid Column

**DOI:** 10.3390/s23084045

**Published:** 2023-04-17

**Authors:** Dumitru-Daniel Bonciog, Mihaela-Ruxandra Lascu, Liliana Mâțiu-Iovan, Valentin Laurențiu Ordodi

**Affiliations:** 1 Measurements and Optical Electronics Department, Politehnica University Timisoara, 300006 Timisoara, Romania; daniel.bonciog@upt.ro (D.-D.B.); mihaela.lascu@upt.ro (M.-R.L.); liliana.matiu-iovan@upt.ro (L.M.-I.); 2 Chemistry and Engineering of Organic and Natural Compounds Department, University Politehnica Timisoara, 300006 Timisoara, Romania

**Keywords:** tissue engineering, Langendorff device, heart discoloration, software application, software optimization, heart decellularization, heart simulation, vibrating fluid column, biomedical images, cardiac biomatrix

## Abstract

This paper presents the validation of a software application to optimize the discoloration process in simulated hearts and to automate and determine the final moment of decellularization in rat hearts using a vibrating fluid column. The implemented algorithm specifically for the automated verification of a simulated heart’s discoloration process was optimized in this study. Initially, we used a latex balloon containing enough dye to reach the opacity of a heart. The complete discoloration process corresponds to complete decellularization. The developed software automatically detects the complete discoloration of a simulated heart. Finally, the process stops automatically. Another goal was to optimize the Langendorff-type experimental apparatus, which is pressure-controlled and equipped with a vibrating fluid column that shortens the decellularization time by mechanically acting directly on cell membranes. Control experiments were performed with the designed experimental device and the vibrating liquid column using different decellularization protocols for hearts taken from rats. In this work, we used a commonly utilized solution based on sodium dodecyl sulfate. Ultraviolet spectrophotometry was used to measure the evolution of the dye concentration in the simulated hearts and, similarly, to determine the concentrations of deoxyribonucleic acid (DNA) and proteins in the rat hearts.

## 1. Introduction

Tissue engineering is a new area of research that was developed because of the absence of transplantable organs. Biomatrices are created through tissue engineering to enable the development and differentiation of numerous cell types [1,2]. In essence, this phase is required to produce a transplantable, three-dimensional, fully recellularized construct. Currently, tissue engineering provides numerous options for decellularization using various physicochemical processes.

Interest and scientific study activities in this area rose following the development of the modified Langendorff infusion system by Ott and his associates [3]. One can conclude that there is a potential for this subject to address unmet medical requirements based on the sparse number of human cases recorded thus far. At present, physicians use these new technologies. A great number of people benefit from this research and solve technical issues.

The potential of rapid graft rejection can be reduced if the decellularization process is completed for the extracellular matrix (ECM) in all cellular components, including DNA [4].

Decellularization must keep the original ECM intact to produce a biomatrix that maintains as much of its mechanical characteristics, biological composition, and ultrastructure as possible [5,6,7,8].

The obtained biomatrix must obey the architecture of the relevant organ (heart, kidney, liver, vascular, etc.). The best matrix is absent of any of its own cells from the organ. It should include the fewest amount of its own cellular components [9,10]. Cells obtained in the laboratory and originating from the person on whom the transplant is to be performed will later be used to populate these acquired matrices [11].

Physical, chemical, and enzymatic methods are combined with decellularization techniques. When decellularizing a tissue or organ, a variety of chemicals are used, mainly chemical or enzymatic detergents, that operate on the tissue and organ.

The lack of a standardized and highly efficient decellularization process for obtaining biomatrices with sufficient three-dimensional structures poses significant challenges for both physicians and biomedical engineers.

All the methods presented offer a high degree of decellularization, but some damage may also occur, which undoubtedly affects the quality of the organ used for transplantation. Immune phenomena are avoided in this way as they are known to be quite severe and often require intensive pharmacological treatments.

Experience has shown that the duration of contact between the decellularizing agent and the organ influences how severe the damage is [12]. Biomedical engineers must develop an algorithm to determine when the decellularization process is completed (when the fewest of the matrix’s own cells are left or when the organ is structurally the least damaged).

This goal has not been achieved. Different techniques have been used for the decellularization process, but most of them have not been performed in real-time (spectrophotometric techniques, different 3D scanning techniques, histological techniques, etc.).

In this study, more efficient approaches are defined with a variety of devices used by many researchers.

The devices developed for this purpose are automation systems that use automated devices such as pressure sensors, optical sensors, peristaltic pump controls, laser scanning, and electronic scanning microscopes.

It can be said that the basic design of the Langendorff device is the basis for the devices that are in use around the world today. Each of these devices has been adapted to the specific perfusion requirements of the organ to be decellularized. The biomedical engineer’s goal is to optimize all the equipment in terms of software and hardware to achieve the desired result. Research is now focusing on the above issues on a global scale.

The decellularization of complete organs for subsequent re-cellularization has received much more attention in the literature. The aim is to obtain functional organs that can reduce the need for allogeneic organ transplantation [13,14,15].

Most of the current systems are developed in research institutes. Only a few commercially available bioreactors designed for decellularization are used by these institutes. They use special equipment and are not yet on the market [16,17,18,19].

The process of decellularization often requires the performance of many phases. Normally, this is carried out manually. A process operator is needed who can perform many manipulation and monitoring tasks. Due to the possible impurities or errors that may be introduced or that occur, we can conclude that there is a risk from both a repeatability and safety perspective.

According to the literature, researchers are also interested in developing and designing different experimental devices that combine decellularization mechanisms. These would allow both large and small organs to be decellularized in the shortest possible time using stable three-dimensional structures of the organs and their biomechanical suitability [20].

In the second section of this paper, we present the construction and validation of an experimental pressure-controlled Langendorff device using a vibrating fluid column. A rapidly oscillating perfusion pressure that exerts a direct mechanical force on cell membranes can accelerate the process of decellularization. Azhim and his team used ultrasound vibration to illustrate this phenomenon [21,22,23,24].

The use of ultrasound in electrochemistry, nanotechnology, food technology, chemical synthesis, and material extraction is currently being pursued by research institutions and companies with expertise in a much wider range of activities [25].

These different methods can accelerate the decellularization process and improve the quality of the resulting scaffolds, which could have a significant impact on how these scaffolds are used in tissue engineering and regenerative medicine [26,27,28].

## 2. Automated Decellularization through Software Optimization

A concept consisting of modeling the decellularization process was proposed, with a low cost to prevent the sacrificing of hearts obtained from rats, based on the gathered needs for testing and verifying the software used.

Therefore, using this technique, the simulated heart provides the transparency of a heart and enables the software algorithm to be tested and modified in real-time, allowing the process to be repeated after any potential errors or any improvement with each test run to meet the requirements of and be prepared for use in a real environment.

This preliminary software program uses a video camera so that the user can see in real-time the heart and simply place it in the decellularization chamber. The software algorithm is used to automate the decellularization of the rat heart. The main purpose of its use is especially for the image acquisition of simulated hearts containing dye.

The software program must include a user interface, often known as a GUI or “Graphical User Interface,” that allows users to interact with it and view activities that are undertaken in real-time in front of the camera that is used. At the same time, it must not only allow the user to enter the time interval in which the images will be acquired but also the possibility of selecting an area where the heart is found for further processing and analysis of the acquired images.

A start button must be created to begin the collecting, processing, and analysis of the biomedical images.

The developed and utilized system must include internal storage for both the processed and captured images. To see how the dye in the simulated heart changed over time, the data that were collected and the results of the image processing process should be saved and represented.

### 2.1. Experimental System

The automation of the decellularization process involves the development of a complex experimental device, which brings together two essential components: the physical part of the system (hardware implementation) as well as the logical part (software implementation).

#### 2.1.1. Hardware Implementation

An experimental device was created to validate the software application that allows obtaining cardiac biomaterials provided with a biomedical image enhancement, processing, and analysis system [29,30].

**Figure 1:**
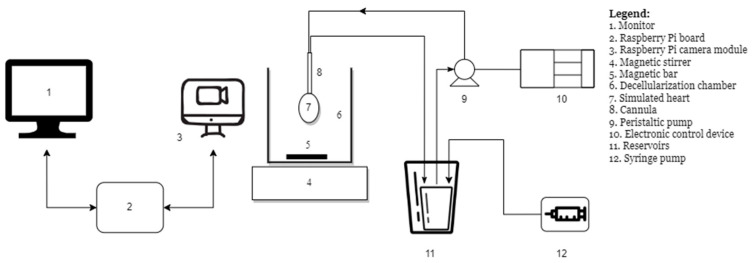
Block diagram of the experimental system.

The block diagram related to this automation system is illustrated in the following Figure 1.

This application was tested using a latex balloon designed to simulate a rat heart. Initially, the simulated heart (Figure 1. 7) was placed in the decellularization chamber (Figure 1. 6). This has enough dye (Azocarmine B dye was used) to give the appearance of a real heart’s opacity.

To retain the same biological parameters as in the real instance, this decellularization chamber included distilled water, and it was homogenized throughout the tests with the use of a magnetic stirrer and bar (Figure 1. 4 and 5). To mimic the decellularization of a real heart, distilled water was injected using a syringe pump for clinical use (Figure 1. 12) at a constant flow rate (50 mL/h) chosen by the researcher. As a result, the dye in the two reservoirs of various sizes (Figure 1. 11), which was gradually diluted with the selected distilled water flow rate, was aspirated using a peristaltic pump (Figure 1. 9) and injected into the model heart.

An electronic control module (Figure 1. 10), originally described by D. Bonciog and their team [31], controlled the peristaltic pump.

Gradually, a color gradient forms that closely resembles how a genuine heart decellularizes, whereby complete discoloration equals total decellularization.

We intended to mimic a heart with this system, test its functionality, and process and analyze biomedical pictures so that, in the future, we may use it to track the progression of the decellularization process in any heart that undergoes this operation.

The “Single-board computer” or “SBC” board Raspberry Pi was used to automate the decellularization procedure.

The Raspberry Pi board is a widely available, reasonably priced alternative compared with a PC, and it serves as the fundamental component of the new hardware design suggested for the automation of this device. Although it is slower than a PC, it offers a variety of benefits such as access to the internet, the ability to create servers and implement educational video and audio games, and automation utilizing its potent CPU and little power requirements [32].

The Raspberry Pi 3 board (Figure 1. 2) (model BCM2837) together with the Raspberry video camera module (Figure 1. 3) were connected to the control component of the experimental device’s hardware to create the final configuration.

The following parts were used to construct the experimental portion of this assembly:

- The Raspberry Pi power supply;
- A 64 GB micro-SD card used not only for the operating system but also for image storage;
- A monitor (Figure 1. 1);
- An HDMI-DVI cable used to connect the monitor to the Raspberry Pi board;
- A keyboard;
- A mouse.

**Figure 2:**
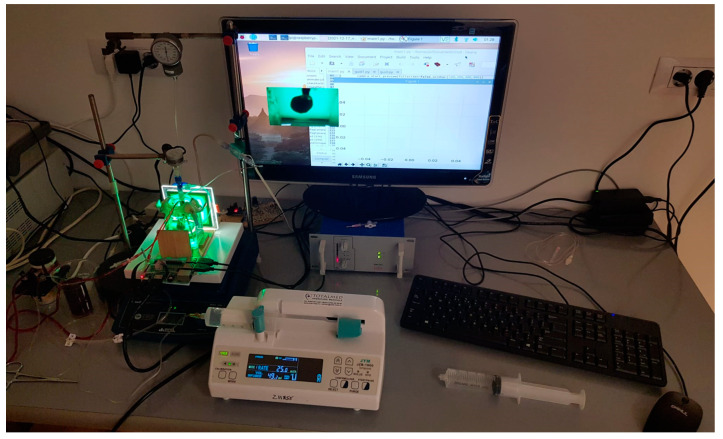
Experimental device to automate the decellularization process.

In Figure 2, the whole experimental device can be seen.

One can see that the decellularization chamber’s front was covered with a square green LED screen. It is well known that myoglobin (red color) in the heart tissue significantly absorbs two colors, red (the color of myoglobin in the structure of the heart tissue) and green radiation, both of which were generated using a light source with a wavelength of approximately λ = 530 nm. Thus, the Beer–Lambert fundamental law of photometry was upheld, enabling a semi-quantitative estimate of the kinetics of the decellularization process. According to the Beer–Lambert rule, there is a linear connection between a solution’s concentration and its absorbance, making it possible to determine a solution’s concentration by observing its absorbance [33].

#### 2.1.2. Software Implementation

The Python programming language was used to create the software application.

The task of a software designer is significantly simplified when compared with using other graphical languages, such as LabVIEW (which is used for the decellularization process), and takes less time to develop when compared with Python.

However, the use of the LabView language is only permitted after purchasing the necessary license. Nevertheless, this licensing fee can vary based on the kind of operating system that is utilized to create the application. In contrast, because Python is a text-based programming language, it takes more time to develop new applications with it than it does with LabVIEW.

Python, on the other hand, is open-source and portable, letting software developers use it on a variety of operating systems without being restricted by licensing rules. Some SBC devices may be used with it as well. Herein, the Raspberry Pi board serves as the hardware architectural control board and is used to operate the developed software program.

For Python, a broad variety of packages and libraries are also readily available, which streamlines the development process. Python is perfect for complicated projects and applications due to its optimization features.

Python is a dynamic scripting language that makes it less ideal for creating programs from the ground up and is largely used for integrating various components. Components are designed to be reusable, and interfaces between them and scripts are well specified.

The optimization of the created software algorithm is used to automatically validate the decellularization process for a simulated heart (containing dye), using comparative ultraviolet spectrophotometry studies for the used dye samples.

This software algorithm allows the user to outline the area of interest for which the acquired images will be processed.

This area is defined by the user at the beginning of each experiment. The area outlined using the graphical user interface buttons does not change during the process and is further used for its processing using the created software algorithm.

Images were acquired at intervals of 5 min during the trial run to test the software application.

The second option, “Stop time,” which controls how many photos are captured, was set to 10 when the software program was constructed. The image is simultaneously sent in real-time to the display. In front of the camera being utilized, this enables the user to position, fix, and frame the heart as effectively as possible. They may also see the mimicked heart’s outline, which is enclosed by four white bars. The processing of the purchased photographs takes place here.

The acquisition of photos was carried out at 5 min intervals during the trial run to test the software application.

To start the procedure, a Start button was included.

The resolution of every acquired photo is 1024 × 768 pixels. When the software application launches, it saves them in a folder that is automatically generated. The experimental data are represented in the folder name. A second folder containing photos with user-framed areas is automatically created by the application in addition to this one. The following stages are used by the software program to process these photos.

The user-defined region is transformed into various shades of gray as part of the first stage of picture processing. The average value of each pixel that makes up the corresponding picture is computed in the second phase. Next, we examine each image’s pixels from a grayscale perspective, wherein white has a value of 255 and black has a value of 0. The arithmetic average between the total number of reported pixels and all the grayscales is used by the program to generate the average grayscale for each image. This “process variable,” which will be referred to as the average value of the pixels in each image, is recorded in a data.txt file and shown on a graph as a percentage.

The values for the visual display are derived from the stored data.txt file. We applied a Gaussian filter to the peaks to smooth them because we could see some variations between the values.

**Figure 3:**
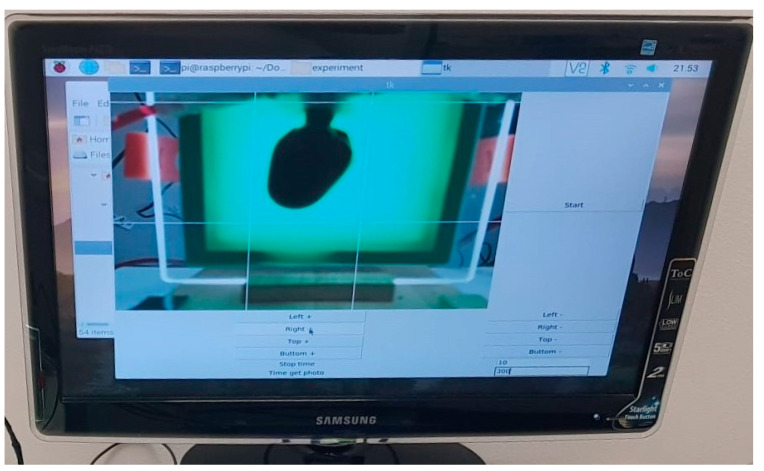
Graphical user interface.

It was observed throughout the experimental studies that the number of photos chosen (the “stop time” constant) was 10 (Figure 3). This means that it is necessary to identify the final point of total discoloration, which is like the total decellularization of a real heart. To consider the process complete, the program compares the most recent 10 values such that there are no variations of less than or more than 3% (the k-value discovered via trials) in the average of the most recent 10 images.

**Figure 4:**
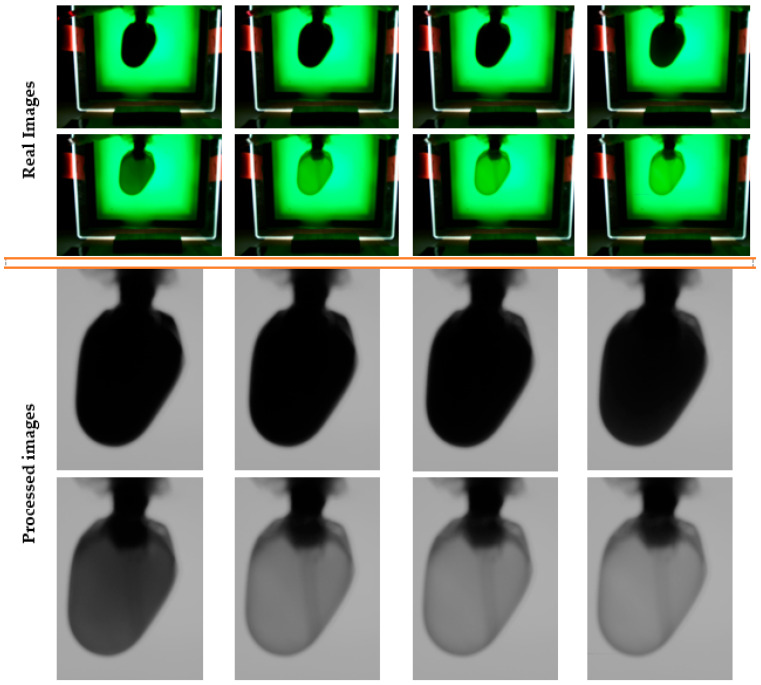
Series of random images acquired from the beginning to the end of the process of discoloration.

All photos were kept in the two appropriate session folders; however, only a small number of randomly selected images from the experiment are shown in Figure 4, along with how the software algorithm processed their appearances.

It was found that the process could be declared finished when the heart was discolored. In this way, the visual appearance of the heart stops changing, and the determined values reach a stationary plateau. In addition, the obtained discoloration times were also normalized to the constant flow rate set on the syringe pump for clinical use for each heart involved in the experiments.

The final moment of discoloration could not be validated with the human eye, making it impossible to find the differences between the last photos, as can be seen in the image series for a randomly chosen experiment (Figure 4).

### 2.2. Experimental Results

#### 2.2.1. Monitoring System

Graphical representations of the values acquired for each image are shown in real-time. Each time a new image is purchased, the chart is updated.

**Figure 5:**
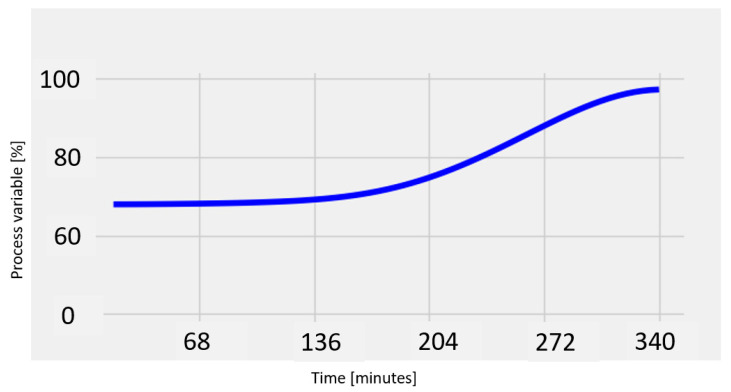
The evolution of the dye during the experiments realized automatically with the software application.

When the simulated heart turns translucent at the end of the procedure, the program automatically ends the procedure and shows an end message to the user. The freshly updated graph is stored in the folder that was first generated automatically. A graph depicts the development of the heart’s simulated color intensity. As seen in the dark blue line in the graph below in Figure 5 (an S-shaped curve), the ultimate outcome demonstrated a sigmoid form [34,35,36].

This procedure took, on average, approximately 340 min, as shown in Figure 5.

To gradually discolorize the simulated heart using the syringe pump for clinical use, distilled water was added at a constant flow rate of 50 mL/h so that at the conclusion of the discoloration process, the software application designed to detect the final moment of discoloration could be used.

#### 2.2.2. Spectrophotometric Test

To monitor the evolution of the dye concentration utilized in this experiment’s procedure, a spectrophotometric technique was employed.

Using an automated micropipette, 10 µL was extracted from the dye reservoir at 30-min intervals. Following the application’s automated shutdown, the discoloration process went on for an additional hour so that more samples could be utilized to verify the application.

A small-volume spectrophotometer was employed for measurements throughout this validation procedure.

The value of the distilled water was measured after each reading of the sample values to reduce the possibility of obtaining inaccurate readings. The background noise of distilled water’s absorbance was determined to be, on average, 0.008 nm.

**Figure 6:**
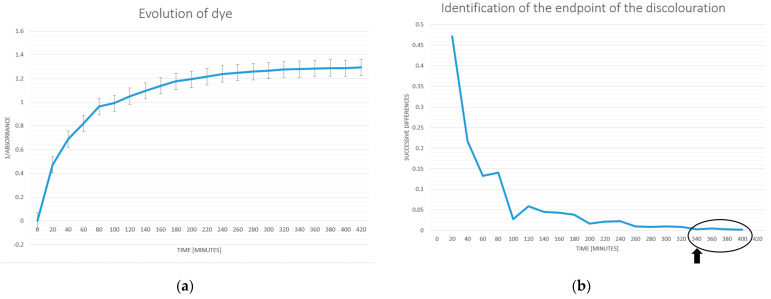
(**a**) Spectrophotometric analysis of dye evolution (average values); (**b**) dye evolution during experiments.

The average values for the evolution of the dye concentration are presented in Figure 6a. The method of consecutive differences is used to determine the point at which the change in the dye concentration becomes unimportant. When the sequential discrepancies reach a fixed minimum value, as shown in Figure 6b, the procedure is said to be finished.

The procedure is finished when the dye concentration drops to a minimum and remains constant, as can be seen starting at 340 min.

We can conclude that the developed software application satisfies the experimental requirements because there are no differences between the duration of the process determined using the proposed software (Figure 5) and the duration determined using the spectrophotometric method that was mathematically processed (Figure 6b).

In conclusion, the developed software is verified and is suitable for use in upcoming investigations of the decellularization procedure using rat heart tissue.

## 3. Experimental Pressure-Controlled Device with a Vibrating Fluid Column

The purpose of this research was to build and implement a hydrostatic Langendorff device with a vibrating liquid column that could superimpose an oscillatory pressure at an order of tens of hertz over the perfusion pressure. In essence, a vertically vibrating column of liquid is produced.

Sodium dodecyl sulfate is also used in this situation as the decellularization solution.

By having a direct mechanical impact on cell membranes, the use of a perfusion pressure that oscillates quickly can quicken the decellularization process.

Applying ultrasonic frequency vibrations served to illustrate this phenomenon in the study by A. Azhim and his team [21,22,23,24]. Applying a low-frequency vibration to the column of decellularizing liquid helps the current investigation determine whether this process takes place.

These various techniques have the potential to speed the decellularization process while also improving the quality of the resultant scaffolds, which might have important consequences for their applications in tissue engineering and regenerative medicine.

### 3.1. Experimental Setup

An instrument consisting of a kind of Langendorff device that is pressure-controlled was designed in this approach.

A pressure oscillation with a frequency of 18 Hz is superimposed over the perfusion pressure using the suggested technology. Basically, a column of vibrating fluid is created, and the related vibrations are then transferred to the rat heart [37].

#### 3.1.1. System Components

The experimental device operates according to Langendorff’s theory. The decellularization solution flow is adjusted with the device to maintain a consistent cardiac perfusion pressure.

The block diagram of this experimental device is shown in the picture below.

**Figure 7:**
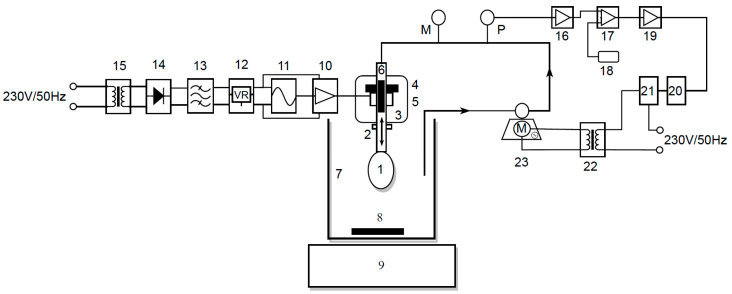
The schematic block of the proposed system.

Below is a table (Table 1) with the legend for the block diagram in Figure 7, which may be used to understand it.

**Figure 8:**
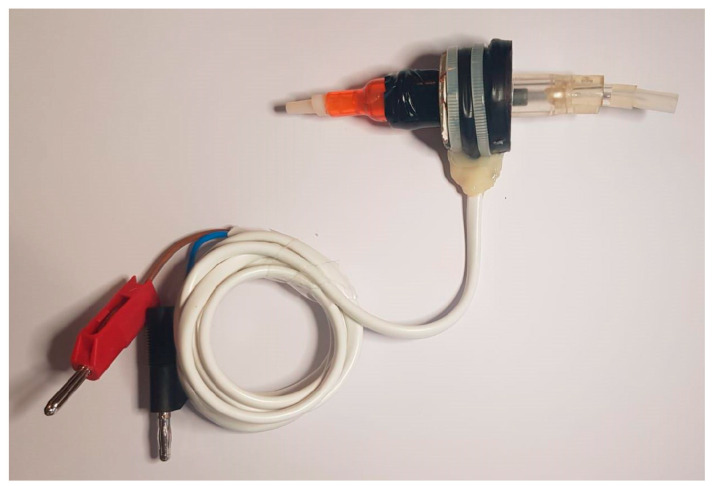
Electromagnetic assembly.

The electromagnetic assembly (Figure 7. 3 and Figure 8) that creates the vibrating column of fluid is linked to the heart (Figure 7. 1), which is ready for decellularization, and this is fastened to the tip of a specific cannula made using AD instruments (Figure 7. 2).

The heart is put through the procedure using a decellularization chamber (Figure 7. 7) that contains a cell lysis solution (SDS, with a concentration in the range of 0.5–1.5%). During the tests, the solution is homogenized using a magnetic stirrer (Figure 7. 9) and a magnetic bar (Figure 7. 8), allowing the composition of the solution to be constant at all points in time and in chamber volume. For gathering samples for biochemical analyses, this step is crucial.

The electromagnetic assembly is made up of a permanent magnet in the shape of a perforated disc (Figure 7. 4) to which a coil with an impedance of Z = 8 is attached (Figure 7. 5). A cylindrical ferromagnetic bar that is covered in Teflon and traversed by a glass tube with a 1 cm diameter is within (Figure 7. 6). Through this glass tube, the SDS solution is pumped via the system through the heart. The heart is in the SDS solution, and this decellularization solution is aspirated from the decellularization chamber with the peristaltic pump. The magnetic field created by the permanent magnet holds the bar in a fixed place so long as the coil is not powered. The coil starts to oscillate when an alternating voltage is applied, and the bar starts to oscillate around the equilibrium point.

The device’s constructive characteristic determines the oscillations’ maximum amplitude, which was measured experimentally with a frequency of 18 Hz at a distance of approximately 9 mm. This maximum amplitude relies on the current intensity and on the frequency of the oscillations.

Following the device’s filling with the decellularization solution, measurements are made. Using a peristaltic pump, the decellularization solution is aspirated from the decellularization chamber (Figure 7. 23). After that, it is returned to the heart via an electromagnetic assembly and cannula.

A cooling approach is adopted, utilizing a fan that is driven at a voltage of 12VDC using a commercial power supply due to the limited heat transfer capabilities of the electromagnetic assembly that were observed during the decellularization process.

A pressure transducer continually measures the perfusion pressure of the heart, which is the pressure on the peristaltic pump’s discharge branch (Figure 7. P). The mechanical pressure gauge also allows for reading it (Figure 7. M).

The automation system combines the pressure transducer amplifier (Figure 7. 16), which amplifies the signal produced by the pressure transducer, and the comparator module (Figure 7. 17), which receives both the amplified signal from the pressure transducer as well as a reference voltage (Figure 7. 18) adjusted from a potentiometer, controlling the operation of the peristaltic pump. The output of this comparator is sent to the solid-state TRIAC–relay (Figure 7. 20), which is utilized to regulate the operation of the peristaltic pump, via power amplifier 2 (Figure 7. 19). The peristaltic pump AC motor (Figure 7. M~) requires a step-down transformer (Figure 7. 22). It is advised to utilize a snubber circuit (Figure 7. 21) designed to safeguard the TRIAC while switching the inductive load (a step-down transformer).

With the aid of power amplifier 1 (Figure 7. 10), the electromagnetic assembly’s power supply is carried out, and this amplifier supplies enough power to cause the ferromagnetic bar to oscillate [38,39].

**Figure 9:**
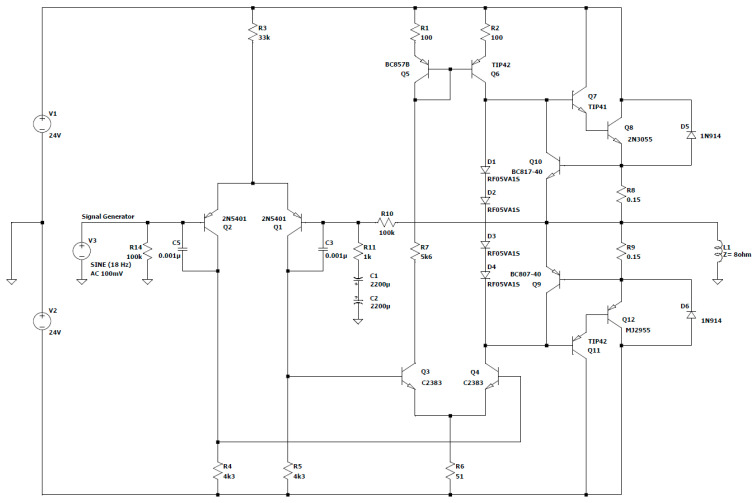
Electrical diagram of power amplifier 1.

The schematic for this amplifier, which does not need any special tweaks and works well, is shown in Figure 9. It was built using inexpensive THDs (through-hole devices), which are easy to obtain.

As can be seen, the electrical components’ values were selected to allow the amplifier to function at a load impedance of Z = 8 Ω and a frequency of 18 Hz.

If an alternating voltage is applied to the coil, the bar will start to move back and forth from its resting point. The highest point that the bar reaches during these movements, known as the maximum amplitude, was determined via the design of the device and was measured to be approximately 9 mm when the frequency is 18 Hz.

The maximum amplitude of the sinusoidal output signal (V_amp_) and the maximum value of the currents (I_elmg_ and I_amp_) in this situation may be computed by taking into account the electromagnet’s voltage, V_elmg_, which is V_elmg_ = 16 VAC:

(1) $$V_{amp}=V_{elmg}\times \sqrt{2}=16\;V\times \sqrt{2}=22.62\;V$$

(2) $$I_{elmg}=\frac{V_{elmg}}{R_{elmg}}=\frac{16\;V}{8\;Ω}=2\;A$$

(3) $$I_{amp}=I_{elmg}\times \sqrt{2}=2.82\;A$$

It was decided to utilize a differential supply voltage, V_alim_ = ±24 VDC, because of the voltage decreases along the route of the inductive load.

(4) $$V_{alim}=V_{amp}+V_{CEsat}+V_{shunt}=22.62\;V+1\;V+(2.8\;A\times 0.15\;Ω)\approx 24\;V$$

This amplifier performs exceptionally well in terms of the specifications for such a basic circuit.

The supply voltage was selected because it is possible to create a 24 V voltage using a variety of power sources that may be more readily available. This amplifier can be powered, for instance, using a 12 V battery with a DC-DC converter that doubles the voltage or using a step-down transformer with a Schottky diode bridge rectifier that delivers the necessary voltage.

This design was dimensioned in resting circumstances while considering the input signal’s amplitude of 0 mV as well as any potential load peaks that could arise during normal operation.

At a typical amplification factor of 20 and a base current of approximately 2.8 A, transistor Q_8_ requires

(5) $$I_{b\;Q8}=\frac{I_{load}}{\beta 1}=\frac{2.8\;A}{20}=140\;mA$$

For transistor Q_7_ to supply an emitter current of 140 mA with a standard amplification factor of 200, a base current is required of approximately

(6) $$I_{b\;Q7}=\frac{I_{b\;Q8}}{\beta 2}=\frac{140\;mA}{200}=0.7\;mA$$

In this case, both transistor Q_9_ and transistor Q_10_ were introduced for the short-circuit protection of the realized amplifier.

As the base current for the two repeater pairs of the emitter in the final stage, Q_7_, Q_8_, Q_11_, and Q_12_ were taken from the second differential stage alternately, with one pair for the positive alternation and the other for the negative, and the quiescent current through the current mirror was chosen to be on average 8–10 times higher.

(7) $$I_{mirror}=10\times I_{b\;Q7}=7\;mA$$

To obtain a symmetrical potential to the ground from the collector of the two transistors Q_6_ and Q_4_ in the middle floor, the choice of resistors R_1_, R_2_, and R_6_ assumes a situation in which the potentials are as close as possible to the emitter respective the base.

(8) $$V_{e\;Q6}={-V}_{e\;Q4}$$

(9) $$V_{b\;Q6}={-V}_{b\;Q4}$$

(10) $$V_{c\;Q6}={2\times V}_{be}=2\times 0.65\;V\approx 1.3\;V$$

(11) $$V_{c\;Q4}={2\times V}_{be}=2\times \left(-0.65\;V\right)\approx -1.3\;V$$

The use of the group of diodes D_1_ ÷ D_4_ at this stage creates a constant voltage drop, approximately equal to 2.6 V, which is necessary to bias the transistors in the output stage. By bringing them into an AB operating class and providing a no-load current of approximately 40 mA–50 mA, the switching distortions from one alternation to the other are considerably reduced. Since the collector current through Q_6_ mirrors the reference current of the current mirror, we can assume that

(12) $$I_{c\;Q5}=I_{oglinda}=7\;mA$$

To obtain the best dynamics on the differential stage (with a wide excursion of the control voltage) the collector potential of Q_5_ must be as close as possible to the supply line.

Arbitrarily choosing a value of V_c Q5_ = 22.6 V, R_1_ can be calculated as follows:

(13) $$R_{1}=\frac{V_{alim}-V_{c\;Q5}-V_{be}}{I_{mirror}\times \left(1+\frac{1}{\beta 2}\right)}=\frac{24\;V-22.6\;V-0.65\;V}{0.007\;A\times \left(1+\frac{1}{200}\right)}\approx 106.60\;Ω=>R_{1}=100\;Ω$$

In this case, the value of resistance R_2_ = R_1_ and V_b Q6_ = V_c Q5_. For the calculation of the resistance R_6_, the double value of the current in the current mirror is taken into account.

(14) $$R_{6}=\frac{V_{b\;Q4}+V_{be}{-V}_{alim}}{{2\times I}_{mirror}\times \left(1+\frac{1}{\beta 2}\right)}=\frac{-22.6\;V-0.65\;V+24\;V}{2\times 0.007\;A\times \left(1+\frac{1}{200}\right)}\approx 53.30\;Ω=>R_{6}=51\;Ω$$

Due to the fact that the emitter of the transistor Q_3_ has the same potential as the transistor Q_4_, its base voltage can also be considered identical, such that V_b Q3_ = V_b Q4_ = −22.6 V, and the resistor R_7_ must withstand the voltage difference related to the mirror current, but without forcing transistor Q_3_ into saturation, a condition imposed by V_c Q3_ > V_b Q3_. Changing the reference in the collector of Q3 gives R_7_:

(15) $$R_{7}<\frac{V_{c\;Q5}+V_{b\;Q3}}{I_{mirror}}<\frac{22.6\;V+22.6\;V}{0.007\;A}<6457\;Ω=>R_{7}=5.6\;kΩ$$

This chosen value is noticeably lower and gives some reserve until transistor Q_3_ saturates. Therefore, this results in a potential for the collector of Q_3_, which has a value of

V_c Q3_ = V_c Q5_ − I_mirror_ × R_7_ = 22.6 V − 0.007 mA × 5600 ohm = −16.6 V (16)

The input stage is of the voltage differential amplifier type, containing the identical transistors Q_1_ and Q_2_ (2N5401). It allows the voltage amplification, with a minimum percentage of distortions, of the input signal.

The amplified signal is taken from the collector of transistor Q_2_ and applied to the base of transistor Q_4_. It is located within the assembly to fulfill the function of the pilot floor.

The dimensioning of the differential input stage is also conducted under symmetry conditions, the base and collector voltages being identical to the null input signal. Thus, the currents through resistors R_4_ and R_5_ can still be considered identical.

Unlike the middle stage in which the current was forced via the power pairs in the Darlington configuration, in the differential input stage, the common current is forced via the base current of transistors Q_3_ and Q_4_.

Considering the typical application factor of β_3_ = 200, R_3_ results:

(17) $$I_{R3}=10\times 2\times \frac{I_{mirror}}{\beta 3}=10\times 2\times \frac{0.007\;A}{200}=0.7\;mA$$

(18) $$R_{3}=\frac{V_{alim}-V_{be}}{I_{R3}}=\frac{24\;V-0.65\;V}{0.0007\;A}\approx 33\;kΩ$$

Taking into account the fact that at rest, the current is divided equally between the two branches of the differential stage and the currents through the resistors R_4_ and R_5_ can be considered identical, the value of the resistors R_4_ and R_5_ can be calculated as follows:

(19) $$R_{5}=\frac{V_{b\;Q3}{-V}_{alim}}{\frac{I_{R3}}{2}\times \left(\frac{\beta }{\beta +1}\right)}=\frac{-22.6\;V-(-24\;V)}{\frac{0.7\;mA}{2}\times \left(\frac{200}{200+1}\right)}=4020\;Ω=>R_{5}=4.3\;kΩ=R_{4}$$

The general amplification of the assembly is given according to the following ratio:

(20) $$Gain=\frac{R_{10}}{R_{11}+2\times \left(\frac{1}{\omega \times C}\right)}=\frac{100\;kΩ}{1\;kΩ+2\times \left(\frac{1}{2\times \pi \times f\times C}\right)}$$

(21) $$Gain=\frac{100\;kΩ}{1\;kΩ+2\times \left(\frac{1}{2\times \pi \times 18\;Hz\times 0.0022\;F}\right)}=99.20;$$

Pairs of complementary Darlington type transistors, such as transistors Q_7_ and Q_8_ (bipolar, NPN type), and Q_11_ and Q_12_ (bipolar, PNP type), are used to create the final stage.

Low frequency is a characteristic of the bipolar transistors used (Q_8_—2N3055 and Q_12_—MJE2955). They work well in such amplification applications and simultaneously provide stability.

Because they are produced in a TO-3 power metal capsule, these transistors achieve a significantly quicker transmission of heat to an aluminum radiator that is made of the same material, which was the criterion for choosing them after reliability and cost optimization.

Two finned aluminum heatsinks separated with an insulating layer were utilized to transport heat away from the device and the remainder of the circuit.

Due to the low heat transfer capabilities reported during the test process (much less compared with the time required for the decellularization process) a substantial cooling method was chosen, using a fan that is powered at a voltage of 12VDC by means of a commercial power supply (230 VAC–12 VDC, 400 mA).

In general, an oscillator or waveform generator is used for every piece of electrical equipment. A source of regular oscillations is required for every measuring equipment or instrument that starts processes or measurements, with the obvious exceptions of signal, function, and pulse generators. Oscilloscopes, digital multimeters, radio frequency receivers, computer peripherals, digital instruments, mobile phones, and many other electronic devices utilize one.

In this case, a Wien bridge oscillator with an operational amplifier (Figure 7. 11) was developed for the input of the power amplifier (Figure 7. 10), and it generates a sinusoidal signal with a constant amplitude of 100 mV.

In this application, Analog Devices’ LT6015 operational amplifier is employed. It is employed in a non-inverting amplifier connection, whose input signal is further linked to the output via a Wien network made up of two pairs—a pair of resistors and a pair of capacitors—in each pair. The oscillation frequency and attenuation are set according to the positive feedback loop formed by this network [40,41].

A pair of diodes are used in the oscillation amplitude limiting circuit of this op-amp Wien bridge oscillator, which is activated when the oscillation amplitude increases and keeps the oscillation point steady. The purpose of this circuit, which is wired to a negative feedback circuit, is to automatically adjust the gain because if it is set too low, the circuit will not oscillate, and if it is set too high, the output signal will be rectangular and have a maximum amplitude.

**Figure 10:**
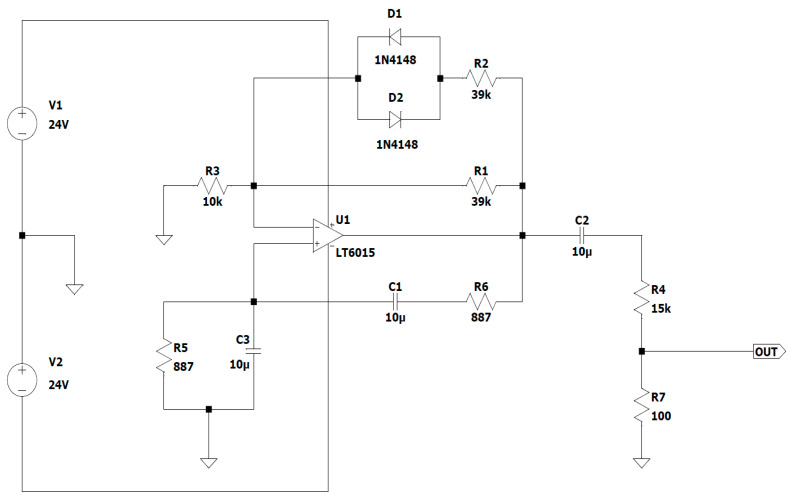
Wien bridge oscillator with operational amplifier.

The Wien bridge oscillator’s circuit diagram, which employed the LT6015 operational amplifier, is shown in the Figure 10.

A band-pass filter is represented by the two phase-shifting circuits that make up the Wien bridge. The reverse phase shift circuit is represented by R_5_ and C_1_, and the forward phase shift circuit by the pair C_3_ and R_6_.

At low frequencies, the forward phase-shifting circuit and the signal output from the bridge, which is connected to the non-inverting input of the AO, phase shift the input signal in front of the output signal, and at high frequencies, the backward phase-shifting circuit and the output signal are out of phase with the input signal.

The phase shift between the input and output voltages is zero at the resonant frequency, and the output voltage peaks when the attenuation is

(22) $$A=\frac{1}{3};V_{out}=\frac{1}{3}V_{in}$$

As shown in Figure 10, R_5_ = R6 = R and C_3_ = C_1_ = C; hence, the resonant frequency may be determined using the following formula:

(23) $$f_{rez}=\frac{1}{2\pi RC}\approx 18\;Hz$$

The values of resistors R_1_, R_2_, and R_3_ were selected to meet the need for voltage gain across the loop (A_O_), which is calculated as the sum of the closed-loop amplifier gain (A_V_) plus the attenuation caused by the feedback circuit (A_R_):

(24) $$A_{O}=A_{v}\times A_{R}=1$$

(25) $$V_{out}=\frac{1}{3}V_{in}\Rightarrow A_{v}=\frac{R_{1}+R_{2}∥R_{3}}{R_{1}}=\frac{10k+\frac{39k\times 39k}{39k+39k}}{10k}\approx 3$$

Choosing the values of the resistors as R_4_ = 15 kΩ and R_7_ = 100 Ω sets the value of the sinusoidal signal amplitude to be approximately 130 mV, which is required for the input of power amplifier 1.

The power supply of the assembly made with power amplifier 1 and the Wien bridge oscillator with the operational amplifier is carried out with the help of a bridge rectifier (Figure 7. 14) with a center-tapped transformer (Figure 7. 15) and a capacitor filter (Figure 7. 13) [42].

**Figure 11:**
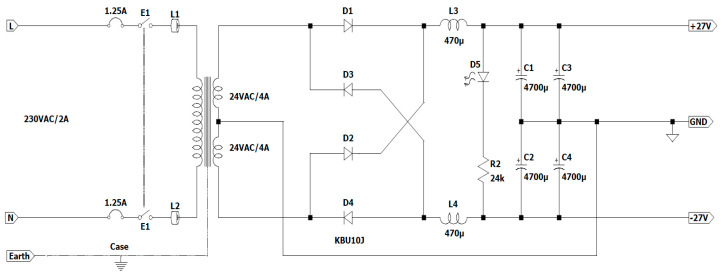
Circuit diagram for a dual power supply.

The Figure 11 shows the schematic that includes these last three electronic blocks.

The transformer that is used is a toroidal type of center-tapped transformer, with a primary voltage of 230 VAC and a secondary voltage of 24 V and 4 A for secondary 1 and secondary 2, respectively.

The value of direct current that has the same calorific impact (the same amount of power used for a load) as the amount of alternating current taken into consideration is known as the effective voltage U_ef_/U_RMS_ (RMS—“root mean square”), according to Storr Wayne [43].

The calculation’s formula is shown below:

(26) $$U_{ef}=\sqrt{\frac{1}{T}\int_{0}^{T}x^{2}\left(t\right)dt}$$

For a pure sinusoidal waveform, this U_ef_/U_RMS_ value will always be

(27) $$U_{ef}=\frac{1}{\sqrt{2}}U_{max}$$

In this case, the maximum voltage is equal to U_max_ ≈ 34 V.

In the scheme in Figure 11, 2 coils with an inductance of 470 µH (L_3_ and L_4_) and resistant to a working current of 4 A were chosen to limit the charging current of the electrolytic capacitors C_1_, C_2_, C_3_, and C_4_ when the voltage is applied. At the same time, the use of two ferrites on a round cable is considered, and L_1_ and L_2_ are mounted on the power cable that is connected to the input of the power amplifier to reduce electromagnetic interference.

A total of 2 1.25 A fuses were placed on the supply lines at the input of the circuit to safeguard it. When the electric current surpasses this value for a certain amount of time, the circuit is interrupted.

To demonstrate the proper operation of electronic circuits, the assembly of D5 and R2 is used as a light indicator.

Next, the voltage drops that appear in this scheme are calculated:

- The maximum forward voltage for the bridge rectifier is

V_D_ = 2 × 1.1 V = 2.2 V (28)

- At a frequency of f = 50 Hz,
  (29) $$X_{L}=2\times \pi \times f\times L=0.148\;\Omega$$
  and produces a voltage drop of 3 A, such that V_L_ ≈ 0.44 V.

- The ripple voltage that occurs when discharging the electrolytic capacitor is

(30) $$U_{rpl}=\frac{0.007\;s\times 3\;A}{4700\;uF}\approx 4.47\;V$$

In this case, the output voltage is

U_out_ = 34 V − 2.2 V − 0.44 V − 4.47 V ≈ 27 V. (31)

Next, this resulting differential voltage is used for the construction of a voltage regulator (Figure 7. 12) made with passive components, with the role of maintaining the constant voltage of the source regardless of the situations that may arise [44].

The use of passive components for the construction of this voltage stabilizer was preferred for the lower construction cost compared with dedicated integrated circuits. This approach was also chosen due to the fact that the operating time of this process is long with a considerable amount of current consumption.

It is also necessary to limit the power dissipated from a series element to 8–12 W for thermal reasons when using this type of stabilizer. This restriction usually results in a limited number of series elements per polarity.

To maintain the stabilization effect even at full load, the input voltage must remain 4–5 V higher than the output voltage, in which case the total power dissipated at a maximum current of 4 A is approximately

(32) $$P_{disip}=5\;V\times 4\;A=20\;W$$

This result allows the use of two power elements in series, each capable of handling a current of 2 A.

According to the datasheet for the operational amplifier, the output current is 14 mA for a 1 V internal voltage drop. A control current for the series elements was chosen to be as low as possible (1 mA), also for thermal reasons. The overall current gain (4000) needed for the series elements to produce a current of 4 A at a command current of 1 mA is derived from this DC current gain.

The series power elements were chosen (these being MJ15022G for the positive alternation and MJ15023G for the negative one, mounted on a radiator, respectively), with an amplification factor of only forty, whereby it was also necessary to add one more element. This particular element is of medium power in the Darlington assembly with the other power elements, with a minimum amplification factor of

(33) $$\beta_{min}=\frac{4000}{40}=100$$

at a collector current of

(34) $$I_{C}=\frac{4\;A}{40}=0.1\;A$$

The maximum dissipated power results as

(35) $$P_{max}=5\;V\times 0.1\;A=0.5\;W$$

Based on the elements that met the imposed condition, it was chosen to use the MJE15030 and MJE15031G transistors, which have a constant amplification factor of 150 in the 0–2 A range.

Separating the load current for the two power elements in series involves the use of shunt resistors to equalize the emitter currents. Their value also determines the current limit at which the protection is activated in case of a possible short circuit.

Considering the maximum load current of 4 A, the activation limit of this protection was arbitrarily chosen to be at a current of 6 A, with the shunt value resulting as follows:

(36) $$R_{shunt}=\frac{0.65\;V}{6\;A}=0.1\;Ω$$

The protection transistor’s maximum collector current is determined via the short-circuit condition and is limited by the op-maximum amp’s output current limit (I_OUT_ = 14 mA) during normal operation.

The calculated voltage is approximate, taking into account the noticeable drop in the rectified voltage at a short-circuit current of 6 A, which is mainly brought on by the transformer’s drooping characteristic. The value of 26 V can be estimated from its technical sheet. Taking into account the transistor in saturation and ignoring the collector–emitter voltage drop, the limiting series resistance can be calculated as follows:

(37) $$R_{limit}=\frac{26\;V-1\;V}{14\;mA}=1.78\;kΩ$$

In this case, the standard value close to the calculated value is $R_{limit}=1.8\;k\Omega$.

A Zenner diode that is supplied with a constant current generator serves as the source of the reference voltage. As a compromise between the constant voltage area and the dissipated power, the diode’s current—of approximately 4 mA—is also arbitrarily selected. The power dissipated from the Zener diode is

(38) $$P_{Zenner}=24\;V\times 4\;mA=0.096\;W$$

The constant current generator picks up the rectified voltage variations, leaving the Zenner diode to be subjected to a relatively constant voltage, thus greatly improving the stability of the stabilized one. While the second diode serves as the constant current generator’s power source, the first diode balances out the emitter–base voltage drop. The actual value of the current is determined via the emitter’s resistance:

(39) $$R_{E}=\frac{0.65\;V}{4\;mA}=162.5\;Ω$$

In this case, the standard value that is closest to the calculated value is 180 Ω.

According to the new value of the current set with the generator, the Zener diode’s power dissipation is as follows:

(40) $$I_{Zenner}=\frac{0.65\;V}{180\;Ω}=3.6\;mA$$

(41) $$P_{Zenner}=24\;V\times 3.6\;mA=87\;mW$$

The two diodes’ current is typically set to be 10–15 times greater than the transistor base’s current. Assuming a low-power transistor with an average amplification factor of 100, it turns out that

(42) $$I_{diodes}=\frac{3.6\;mA\times 15}{100}=0.54\;mA$$

The voltage drops in the elements involved in the stabilization chain are added to determine the minimum input voltage. Thus, using the operational amplifier, it is possible to calculate the voltage drop across the shunt as well as the base–emitter voltage drops of the series elements and the voltage drop across the series output resistance:

(43) $$V_{shunt}=0.1\;Ω\times 2\;A=0.2\;W$$

(44) $$V_{be}=2\times 0.65\;V=1.3\;V$$

(45) $$V_{Rlimit}=1\;mA\times 1.8\;kΩ=1.8\;V$$

The value of the resistance inserted with the 2 diodes can be estimated as follows:

(46) $$R_{D}=\frac{27\;V-2\times 0.65\;V}{0.54\;mA}=47.59\;kΩ$$

The standard value in this situation is 47 kΩ.

The maximum current for a voltage peak, arbitrarily chosen to be 40 V, can be calculated as follows:

(47) $$I_{max}=\frac{40\;V-2\times 0.65\;V}{47\;kΩ}=0.82\;mA$$

Additionally, the transistor’s maximum power dissipation when its base current is absent is as follows:

(48) $$P_{Tmax}=(40\;V-0.65\;V-24\;V)\times 3.6\;mA=55\;mW$$

**Figure 12:**
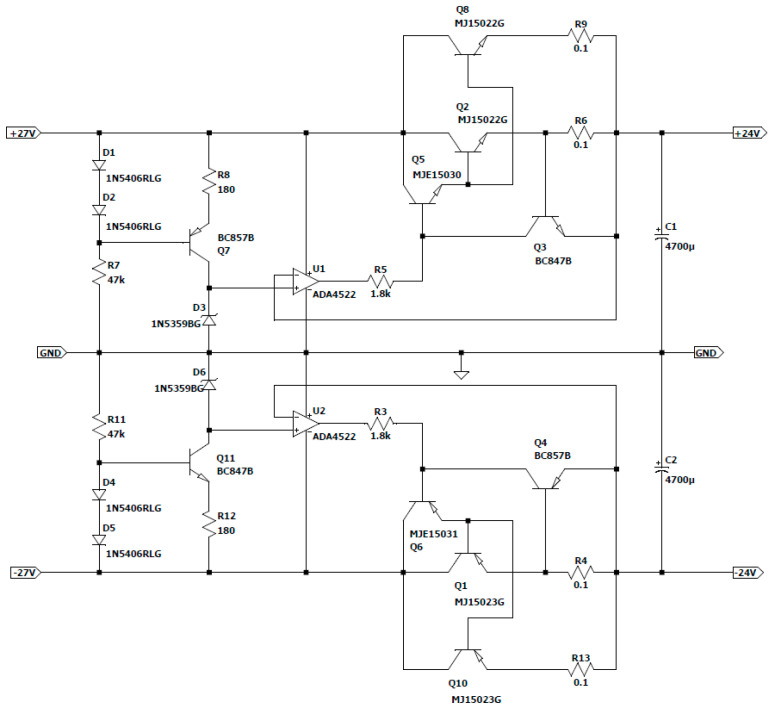
Voltage regulator.

The following Figure 12 shows the regulator design that was created.

#### 3.1.2. Experimental Protocol

Decellularizing the hearts of six Sprague Dawley rats weighing between 300 and 400 g was used to test and validate the experimental device in the Centre for Gene and Cellular Therapies in the Treatment of Cancer at the OncoGen Research Institute in Timisoara.

Hearts were obtained in accordance with both international legislation (The Guide for the Care and Use of Laboratory Animals issued by the National Institutes of Health, no. 85-23) and the guidelines of the Ethics Commission at the UMF “Victor Babeş”, Timișoara. Sevoflurane-infused VIMA general anesthesia was used in every instance. For the induction in a dedicated room, 8% sevoflurane in oxygen was used, and then 3.5% sevoflurane was administered through a face mask to keep the rats under anesthesia until the hearts were removed.

In the first stage, the abdominal cavity of each rat was opened, and 100 IU/Kg of sodium heparin was injected into the inferior vena cava. The thoracic cavity was opened after approximately four to five minutes by removing the ribs, and the heart was swiftly removed along with a piece of the aorta. The cord and a 1 cm long piece of the aorta were perfectly segregated after being put into a Petri dish filled with a cold solution of physiological sodium chloride that had been heparinized [45,46].

In the next step, the heart of each rat was then further treated by fixing it with a silk surgical thread to the cannula of a Langendorff-type device, and it was then infused for 600 min with a 1.5% SDS solution [47].

In the experimental device, there was a 120 cm^3^ volume of the solution.

The contents of nucleic acids and proteins in the decellularization solution were assessed using 30 µL samples collected every 30 min from the decellularization chamber (Figure 7. 7). The UV spectrophotometric technique was used to make these determinations. At a pH of 7, the proteins’ maximum absorptions occur at 280 nm. Because nucleic acids exhibit a maximum absorption at 230 nm under identical circumstances, the 2 analytes can be measured concurrently without being separated [48]. The instrument of choice was a NanoDrop ND-1000 computerized microspectrophotometer. The measurements were made using the device without the need for additional reagents, and the findings are shown in ng/µL for nucleic acids and in mg/mL for proteins.

### 3.2. Experimental Results

#### 3.2.1. Monitoring System

**Figure 13:**
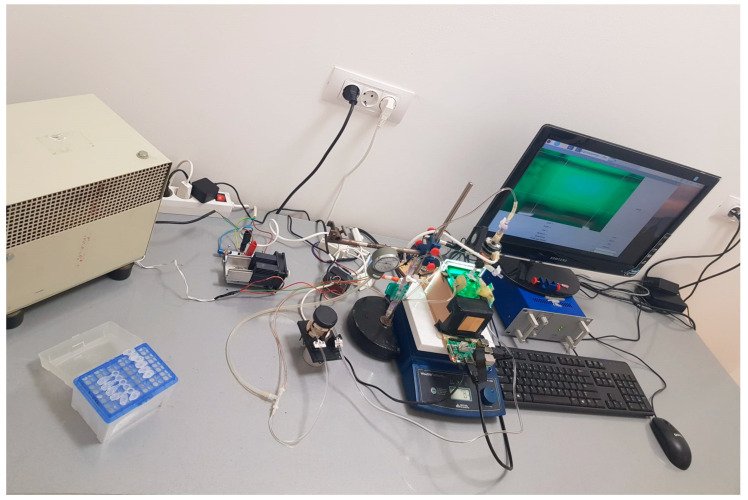
Improved experimental device for automating the decellularization process.

The entire experimental assembly can be seen in Figure 13 below.

In these experiments, images were acquired at 10 s intervals (the “Time get photo” was set to 10 s), and the “stop-time” parameter was kept at 10. It was chosen to use a shorter time interval for creating a solid database of biomedical images from the in vitro decellularization experiments.

The rat hearts were divided into two groups for this study:

- Lot A (n = 3 hearts), which used an experimental device with a vibrating fluid column to superimpose an oscillating hydrostatic pressure with a frequency of 18 Hz over the perfusion pressure;
- Lot B (n = 3 hearts), which served as the control group for the validation of the experimental results. Herein, the vibrating fluid column is off/not powered.

Below, Table 2 displays these two experimental protocols.

**Figure 14:**
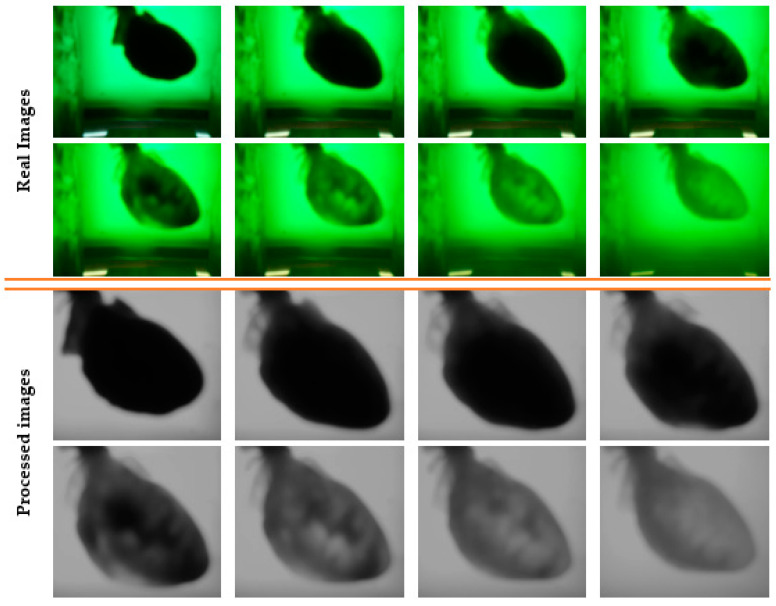
Series of random images acquired from the beginning to the end of the decellularization process.

From the beginning until the end of an experiment, the kinetics of decellularization could be observed, as well as the processing method employed with the software program described below in Figure 14.

Both the control lot (E2) and the first lot (E1) given the vibrating column had their decellularization progress monitored in real-time.

**Figure 15:**
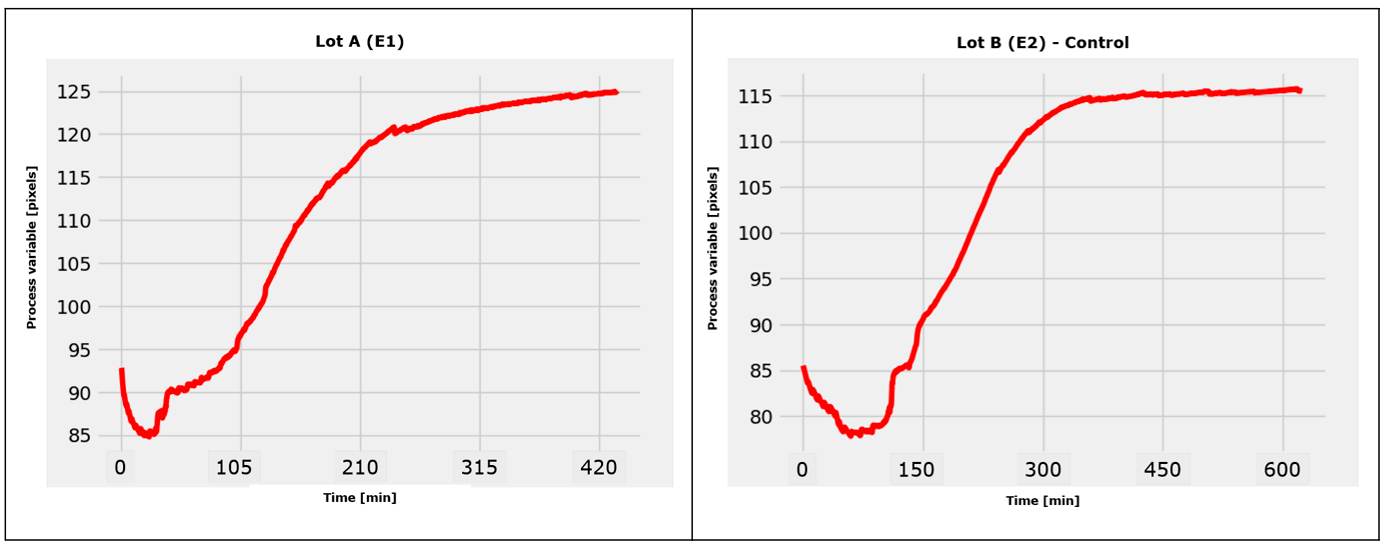
Dependence of the “process variable” on time for lot A and lot B.

The results are shown as average values in the section below in Figure 15.

As can be seen, in the case of the control lot, the monitoring system of the developed software program recognized the final moment of the decellularization process at approximately 600 min. Nevertheless, at 420 min, it is simple to determine the effectiveness of decellularization in the event of applying liquid column vibrations.

In this instance, we can say with confidence that the employment of a vibrating fluid column as a decellularization tool demonstrated good results in lowering the decellularization time, which was lowered by approximately 30% (this percentage was obtained using the data in Figure 15).

Comparative UV spectrophotometry investigations were carried out to confirm these automated outcomes of the software program and to establish the level of precision in the quality of the decellularized scaffolds.

#### 3.2.2. Spectrophotometric Test

The decellularization solution has a volume of 120 cm^3^ in this experimental equipment.

Using 30 µL samples, which were manually taken every 30 min during the decellularization process, the concentrations of nucleic acids and proteins in the decellularization fluid were determined. Each sample obtained a number, and later, it was placed in a box, which was stored in a freezer, until the experiment was performed with the NanoDrop ND-1000 spectrophotometer.

The UV spectrophotometric method was employed to obtain the samples’ concentration values of nucleic acids and proteins.

**Figure 16:**
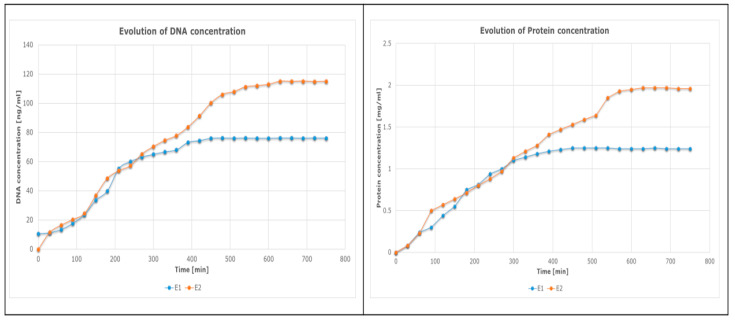
DNA and protein concentration evolutions (mean values).

**Figure 17:**
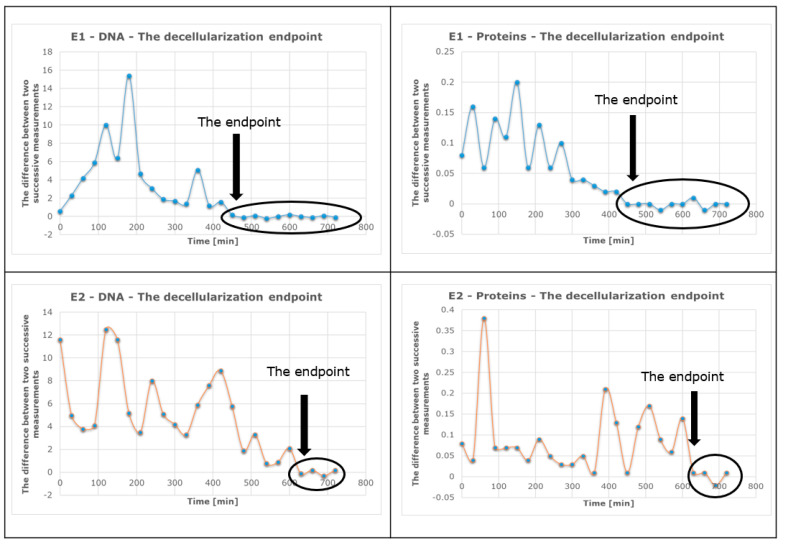
Determining the decellularization endpoint for each experiment.

The outcomes of using the suggested experimental techniques are shown in the section below. The graphical method (Figure 16) was used to identify the so-called plateau of the concentration curves or the point at which the concentration of an analyte (either nucleic acids or proteins) reaches a stationary value, while the successive difference method (Figure 17) may also be used to pinpoint the precise value at which a kind of concentration (of DNA or proteins) starts to become important by recording a minimum and constant value.

The figure below illustrates the concentration curves of both analytes (proteins and deoxyribonucleic acid) over the course of the experiments. The graph shows how the concentrations of the analytes changed over time, each reaching a plateau at a certain point. The plateau values can be used as indicators of the completion of the decellularization process.

The control group (E2) is slower at reaching a stationary value (Figure 16: DNA ≈ 118 ng/mL; protein ≈ 1.9 mg/mL) compared with the vibrating group (E1) (Figure 16: DNA ≈ 79 ng/mL; protein ≈ 1.3 mg/mL); however, the plateau value of E2 is much higher than that of E1.

These values were provided using the spectrophotometric method. These different values do not indicate that there are proteins/DNA left because we have reached the maximum decellularization plateau.

This plateau value reached a stable value much faster for E1 (when vibration was applied) than for E2 (when no vibration was provided).

The hearts were completely decellularized in both cases, the difference being in the time required for decellularization.

A mathematical algorithm that calculates the differences between two consecutive measurements for each experiment was used to determine the final time of decellularization. The identification of the decellularization endpoint for each experiment using this algorithm is shown in Figure 17.

The decellularization process endpoint for each experiment was determined using a mathematical algorithm that calculates the differences between two consecutive measurements.

The effectiveness of decellularization was compared between two situations: when liquid column vibrations were applied (E1) and when they were not applied (E2), as shown in Figure 17.

**Figure 18:**
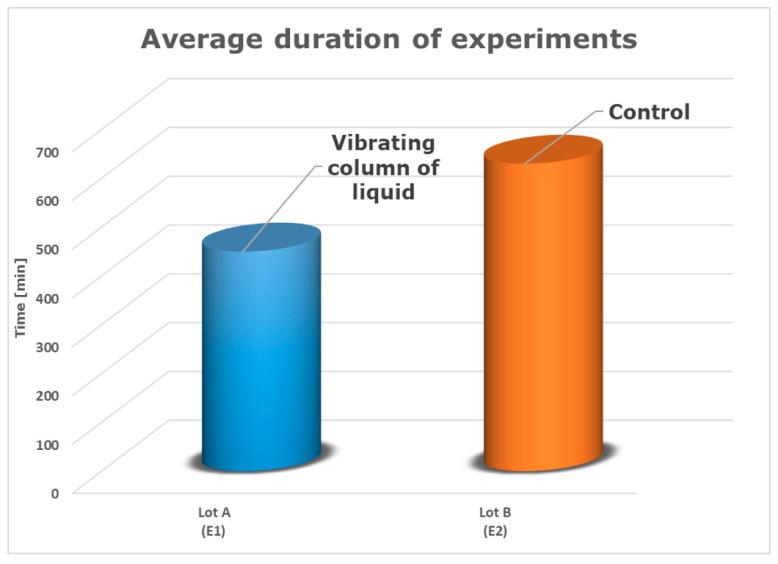
The typical length of each experiment (comparative study).

Additionally, Figure 18 presents a comparison of the average length of each experiment.

In the case of lot B–E2 (control), it can be observed that the final moment of the decellularization process occurred after 630 min. For lot A–E1, the DNA/protein concentration became constant after 450 min of operation with the vibrating fluid column.

Based on the information provided using the spectrophotometric method, it can be concluded that the use of the device with a low-frequency and constant-amplitude vibrating fluid column reduces the decellularization time for a rat heart by approximately 29%. This finding is significant and demonstrates the potential of this device to improve the efficiency of the decellularization process. This could be a promising approach for accelerating the decellularization process in rat hearts, ultimately leading to faster and more efficient decellularization for tissue engineering applications.

## 4. Conclusions

In this work, the discoloration process in a simulated heart was used to validate the software application’s optimization and to determine the final decellularization moment in rat hearts and automate the procedure. This was realized with the help of a vibrating fluid column.

In this study, we optimized the software algorithm that was created to automatically evaluate the discoloration process in simulated hearts. We started with a latex balloon into which we added enough dye to give it the opacity of a real heart. We saved ourselves from sacrificing rat hearts while we developed, modified, improved, and tested the original prototype software algorithm. The process of complete discoloration corresponds to complete decellularization. The developed software automatically detects complete discoloration in a simulated heart, and the process stops automatically.

Another goal was to optimize the entire experimental apparatus, consisting of a pressure-controlled Langendorff-type device equipped with a vibrating liquid column that shortens the duration of decellularization by directly exerting a mechanical effect on cell membranes. Therefore, we developed an electromagnetic assembly, fixed at the level of the cannula, which causes the oscillating movement of the liquid column with a low frequency (in this case, 18 Hz) and a constant amplitude. To operate this electromagnetic assembly, we designed and built a power amplifier, a power supply for the system components, and an oscillator/signal generator so that researchers or doctors could plug the power cable into a socket to use this facility.

We performed control experiments with the designed experimental device and the vibrating fluid column with different decellularization protocols for the rat hearts. In this work, a common solution based on sodium dodecyl sulfate was used. By using ultraviolet spectrophotometric measurement of the evolution of the dye concentration in the simulated heart and also determining the concentrations of deoxyribonucleic acid (DNA) and proteins in the rat hearts, this device was validated.

The experimental device that was developed proved to be very effective in reducing the time required for decellularization and ensuring consistency in the process. The automated function of the device, which ensures capturing the final moment of decellularization in a heart, is also a significant advantage, as it eliminates the need for manual sampling and reduces the risk of human error.

The results of this study suggest that the link between the experimental device and the software algorithm provides an efficient and reliable method for decellularizing rat hearts. This approach could have significant implications for tissue engineering and regenerative medicine, whereby decellularized tissues and organs can be used as scaffolds to produce functional replacement tissues and organs.

The current limitations of this research are related to the fact that only one type of cardiac tissue was used (rat cardiac tissue), which may limit the generalization of the results to other animal species or to human cardiac tissue. Further investigation could involve testing the efficacy of the method on different types of cardiac tissue and on human cardiac tissue to broaden the scope of this research. Additionally, exploring new decellularization technologies or methods could potentially improve the results and accelerate the decellularization process.

Furthermore, it should be noted that the complete impact of decellularization on the structure and function of the myocardium is still not fully understood. The integration of artificial intelligence and nanotechnology may improve the efficiency and precision of the decellularization process. These technologies were not utilized in this study.

Future directions for research efforts could include expanding this study to include other types of tissues or organs and integrating artificial intelligence, machine learning techniques, and nanotechnology into the decellularization process to improve the results [49,50]. Improvements in the evaluation and monitoring methods of the decellularization process could also be considered to increase the precision and reliability of the procedure.

## Figures and Tables

**Table 1:** Block diagram legend.

Nr.	Name	Nr.	Name
1	Heart	14	Bridge rectifier
2	Cannula	15	Center-tapped transformer
3	Electromagnetic assembly	16	Pressure transducer amplifier
4	Permanent magnet	17	Comparator module
5	Coil	18	Reference voltage
6	Ferromagnetic bar	19	Power amplifier 2
7	Decellularization chamber	20	Solid-state relay
8	Magnetic bar	21	Snubber circuit
9	Magnetic stirrer	22	Step-down transformer
10	Power amplifier 1	23	Peristaltic pump
11	Wien oscillator	M~	Peristaltic pump AC motor
12	Voltage regulator	M	Mechanical pressure gauge
13	Capacitor filter	P	Pressure transducer

**Table 2:** Experimental protocols.

	Vibrating Fluid Column
Lot A (E1)	Yes
Lot B (E2)	No (Control)

## Data Availability

Not applicable.
